# Saliva Ontology: An ontology-based framework for a Salivaomics Knowledge Base

**DOI:** 10.1186/1471-2105-11-302

**Published:** 2010-06-03

**Authors:** Jiye Ai, Barry Smith, David T Wong

**Affiliations:** 1 University of California at Los Angeles School of Dentistry and Dental Research Institute, 73-017 Center for Health Sciences, 10833 Le Conte Avenue, University of California, Los Angeles, CA 90095-1668 USA; 2 Department of Philosophy, 135 Park Hall, University at Buffalo, Buffalo, NY 14260 USA

## Abstract

**Background:**

The Salivaomics Knowledge Base (SKB) is designed to serve as a computational infrastructure that can permit global exploration and utilization of data and information relevant to salivaomics. SKB is created by aligning (1) the saliva biomarker discovery and validation resources at UCLA with (2) the ontology resources developed by the OBO (Open Biomedical Ontologies) Foundry, including a new Saliva Ontology (SALO).

**Results:**

We define the Saliva Ontology (SALO; http://www.skb.ucla.edu/SALO/) as a consensus-based controlled vocabulary of terms and relations dedicated to the salivaomics domain and to saliva-related diagnostics following the principles of the OBO (Open Biomedical Ontologies) Foundry.

**Conclusions:**

The Saliva Ontology is an ongoing exploratory initiative. The ontology will be used to facilitate salivaomics data retrieval and integration across multiple fields of research together with data analysis and data mining. The ontology will be tested through its ability to serve the annotation ('tagging') of a representative corpus of salivaomics research literature that is to be incorporated into the SKB.

## Background

Saliva (oral fluid) is an emerging biofluid for non-invasive diagnostics used in the detection of human diseases. The need to advance saliva research is strongly emphasized by the National Institute of Dental and Craniofacial Research (NIDCR), and is included in the NIDCR's 2004-2009 expert panel long-term research agenda [1]. The ability to monitor health status, disease onset, progression, recurrence and treatment outcome through non-invasive means is highly important to advancing health care management. Saliva is a perfect medium to be explored for personalized individual medicine including diagnostics, offering a non-invasive, easy to obtain means for detecting and monitoring diseases. Saliva testing potentially allows the patient to collect their own saliva samples at home, yielding convenience for the patient and savings in health costs, and facilitating multiple sampling. Specimen collection is less objectionable to patients and easier in children and elderly individuals.

Due to these advantages, finding biomarkers in saliva for the detection of serious illnesses such as cancers has been on the national healthcare agenda for several years, and the National Cancer Institute has accordingly recognized saliva as a promising source for cancer biomarkers [2]. A mandate in the Government Performance Report Act (GPRA) report is that by year 2013 proof of principle will be obtained for the ability of saliva to monitor health and diagnose one systemic disease [3].

For the past six years the UCLA salivaomics research group has developed proteome, transcriptome, microRNA, metabolome, genome, microbiome, and point-of-care salivary diagnostic technologies. These research resources have proved highly valuable to basic and translational research groups around the world.

Sjögren's syndrome (SS) is an autoimmune disease that affects exocrine tissues, especially salivary glands and lacrimal glands. The autoimmune-mediated damage of the salivary and lacrimal glands leads to a decrease in the production of saliva and tears and to the development of dry mouth and dry eyes. Without the lubricating and protective functions of saliva and tears, the oral and ocular surfaces are subject to infections and discomfort leading to significantly reduced quality of life. The disease can present either as primary Sjögren's syndrome (pSS), when no other autoimmune diseases are present, or as secondary Sjögren's syndrome (sSS), which involves co-presence of some other autoimmune diseases. Sjögren's syndrome is one of the most common autoimmune disorders in the US, with an estimated prevalence of ~4 million people, affecting primarily women (in a ratio of 9 to 1). In addition, pSS patients have a 40-fold higher risk of developing malignant lymphoma than the general population.

The rapid development and maturity of the genomics field has led to the emergence of other omics studies, such as proteomics and transcriptomics, which are now being implemented widely in studies of human disease. Clearly, mining the data from multiple omics studies can provide deeper insight into the workings of biological systems than can be obtained from any single omics study. Omics databases such as PharmGKB (the Pharmacogenomics Knowledge Base [4]) and EPO-KB (the Empirical Proteomic Ontology Knowledge Base [5]) serve as important resources for the emerging discipline of systems biology as applied to the understanding of pathogenesis in humans and in model organisms. However, systematic management and analysis, and above all the integration, of omics datasets remains challenging, and the problems are compounded when these datasets need to be integrated with data of other sorts, including diagnostic data obtained by clinicians.

As a computational and informatics infrastructure that can permit global exploration and utilization, the Salivaomics Knowledge Base (SKB) is being created by aligning (1) the saliva biomarker discovery and validation resources at UCLA with (2) the ontology resources developed by the OBO (Open Biomedical Ontologies) Foundry [6], including the new Saliva Ontology (SALO) that is described in this communication.

**Figure 1:**
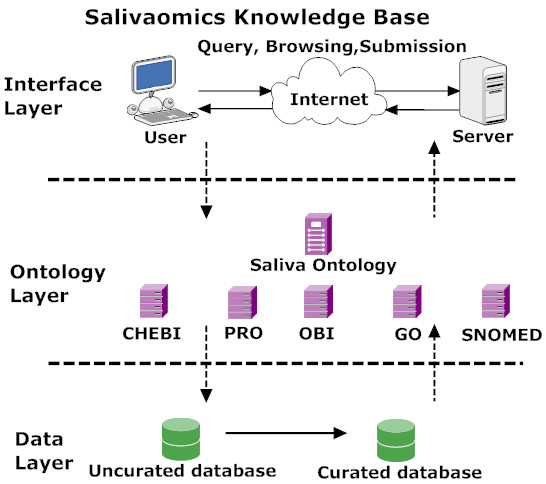
**The SKB architecture**. The SKB is based on a three-layer architecture. Data sources are stored in the data layer. In the ontology layer data elements from these data sources are mapped to nodes in controlled vocabularies. The interface layer allows query, browsing and submission. For instance, the interface layer receives user requests, submits queries to the data layer through the ontology layer and obtains corresponding results. The three layers connect data from consumers (users submitting query) with data providers (data sources) via ontologies.

The Salivaomics Knowledge Base (SKB; http://www.skb.ucla.edu/) is a data repository, management system and web resource constructed to support human salivary proteomics, transcriptomics, miRNA, metabolomics and microbiome research. The SKB will provide the first web resource dedicated to salivary omics studies and will contain the data and information needed to explore the biology, diagnostic potentials, pharmacoproteomics and pharmacogenomics of human saliva. At the same time it will allow a systems approach to the utilization of salivary diagnostic technology for personalized medicine applications. It has an effective information retrieval system and carefully designed data format and employs an open data model. Figure 1 shows the SKB's three-tier service oriented architecture with a Data Layer, Ontology Layer and Interface Layer.

## Results and Discussion

**Figure 2:**
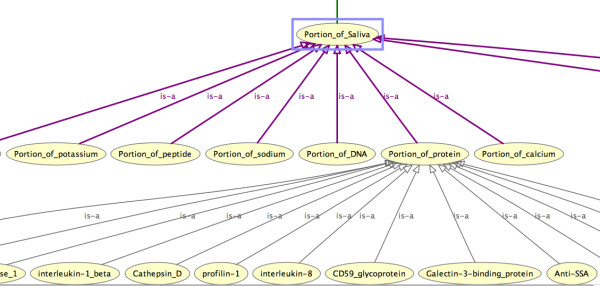
**A fragment of SALO in its current form**.

We are creating the Saliva Ontology (SALO; http://www.skb.ucla.edu/SALO/) as a consensus-based controlled vocabulary of terms and relations dedicated to salivaomics and to saliva-related diagnostics. Figure 2 displays a fragment of SALO in its current form.

### SALO

The Saliva Ontology (SALO) is being created through cross-disciplinary interaction between saliva experts, protein experts, diagnosticians, and ontologists. To aid development and testing of SALO, we are incrementally developing a corpus of saliva-relevant literature in SKB to assist in identifying core terms, synonyms and definitions for inclusion within the ontology, and to provide examples of usage and links between SALO content and the corresponding items through their PubMed identifiers. In this way a growing body of semantically enhanced web-enabled literature will be created within the SKB to support future research. Additional resources upon which we will draw in populating and validating SALO will include the results of experiments in data- and text-mining using the ontology, and cross-linking to existing ontologies and terminology resources involving treatment of saliva-relevant phenomena. We will also identify and represent within SALO relationships to saliva-relevant types represented in ontologies such as Gene Ontology (GO) [7], the Protein Ontology (PRO) [8], Ontology for Biomedical Investigations (OBI) and Chemical Entities of Biological Interest (CHEBI) [9] and also provide links to corresponding SNOMED CT terms where available. To facilitate the maintenance of SALO and its use in literature curation, text-mining tools such as GATE http://gate.ac.uk/ and the Python Natural Language Toolkit are being used.

SALO will be a public domain resource and entirely web-based. Each term in the ontology will have its own URL which will point to a webpage providing definitions, PubMed sources, references to annotations in SKB and to external databases. The goal of the SKB is then to integrate, store, organize and manage all saliva-relevant experimental data annotated and connected through SALO and its associated ontologies. SKB will include also data about saliva-related experiments, which will be captured using the Ontology for Biomedical Investigations (OBI) in tandem with the SALO ontology.

### SALO Development

In order to build on the solid foundations of prior work on biomedical ontologies and on the associated software tools and ontology application techniques, we will work with the Open Biomedical Ontologies (OBO) Foundry, and with the developers of OBO ontologies such as GO, PRO, CHEBI, and OBI in order to ensure conformity with current best practices and to guarantee non-redundant development. We follow the principles of the OBO Foundry in constructing SALO in order to ensure that SALO is semantically interoperable with the other OBO ontologies[6].

### Salivaomics Standard Literature Corpus

We have access to full-text articles of the biomedical journals from PubMed until 2009 and are using these as our primary resource in creating the Salivaomics Standard Literature Corpus (draft version here: http://www.skb.ucla.edu/SSLC/). First we created a list of representative saliva literature items in collaboration with the following scientific leaders (in alphabetical order by last name) in saliva research:

- Arie V. Nieuw Amerongen (Free University and University of Amsterdam, The Netherlands)

- Bruce Baum (National Institutes of Health/NIDCR)

- William Giannobile (University of Michigan)

- James Melvin (University of Rochester)

- Nelson Rhodus (University of Minnesota)

- Charles Streckfus (The University of Texas Health Science Center at Houston)

- Arjan Vissink (University Medical Center Groningen, The Netherlands)

- Stephen Wotman (Case Western Reserve University)

- Chih-Ko Yeh (University of Texas Health Science Center at San Antonio).

31 literature items for inclusion were then selected on the basis of numbers of citations. Some of the articles in the corpus are specifically related to the Sjögren's syndrome test-case. This corpus now forms a constituent part of the SKB, where the SALO ontology, in tandem with GO, PRO and other ontologies, is being applied to create semantically enhanced versions of the initially selected representative articles through a process of manual curation.

In an interesting positive feedback effect, the process of annotating the corpus will in addition bring benefits to the process of development of the SALO ontology itself, since it will help us to identify gaps in the ontology discovered in the process of curation. Where an expression that is relevant to saliva research is used in the corpus but is not already present in SALO, a new ontology term will be proposed and then either added to SALO or submitted to one of the other relevant ontologies for inclusion. In this way, the comprehensiveness of SALO can be incrementally achieved relative to the state of the art in salivaomics research at any given stage, and in a way which ensures the alignment of SALO with other major ontologies.

**Figure 3:**
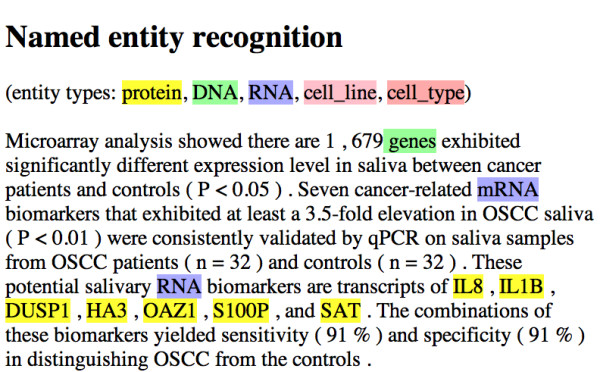
**A result of entity chunking recognition for the text of one sample article**.

In addition to the manual curation of the saliva corpus, we are also using text-mining algorithms to the 23,467 PubMed articles identified as containing occurrences of the term "saliva" in their title or abstract. Specifically we are applying information extraction algorithms for segmentation, part-of-speech tagging, semantic labelling, and entity and relation chunking recognition to these articles. Figure 3 displays the result of entity chunking recognition applied to the text of one sample article.

This strategy, too, will identify additional terms needed in the Saliva Ontology or in the related ontologies with which SALO must interoperate. We will thus pursue both manual and automatic processes for annotating the literature in our corpus, amending SALO incrementally, and also submitting necessary amendment requests to related ontologies as we proceed.

### SALO Resources

**Figure 4:**
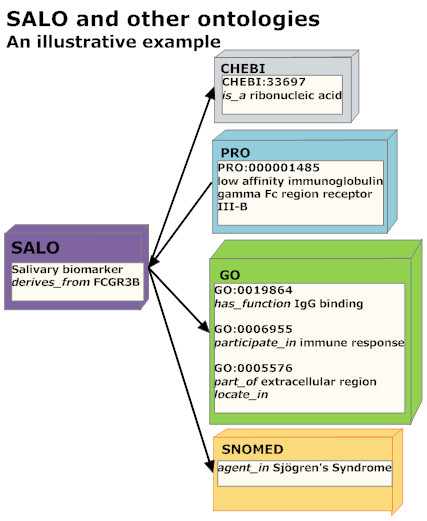
**Links between SALO and other ontologies**.

The Saliva Ontology consists of both hierarchical (*is_a*) structures supporting saliva-domain taxonomies and additional relationships (such as *part_of, derives_from, has_function*) taken from the OBO Relation Ontology [10]. Figure 4 illustrates some illustrative examples of links between SALO and other ontologies.

SALO is a formal ontology created using the W3C standard Ontology Web Language (OWL 2.0). The public release OWL versions of the ontology will be published in the NCBO BioPortal. For quality assurance and dissemination purposes, parallel versions will be created (using standard conversion software) in the OBO format that is used by the GO Consortium and still favored by many biologists. These OBO versions will be submitted for inclusion in the OBO ontology library.

SALO will employ the OBO Foundry principles http://www.obofoundry.org/crit.shtml in its development. This will help to ensure that the ontology is open, well-documented, specific to the domain (saliva, including salivaomics), and that it works well with other biomedical ontologies. The principles require that both logical and natural language definitions are provided for each term in the ontology, and also that links between terms are asserted using relational expressions which have been logically defined in a way to the OBO Relation Ontology [10]. Cross-ontology reasoning is thereby supported by the definitional structure of the ontologies involved.

### Ontology of the Salivary Markers for Sjögren's Syndrome (SS)

SALO will be tested specifically in light of its capacity to meet the ontology needs for managing data derived from research on the use of a salivary genetic marker for Sjögren's syndrome (SS).

In results of a complete screening for the TRIM21 gene in patients with primary SS are presented [11], together with results of a gene association study. A single-nucleotide polymorphism (SNP) in intron 3 was found to be strongly associated with the presence of anti-Ro 52 kd autoantibodies in primary SS.

**Figure 5:**
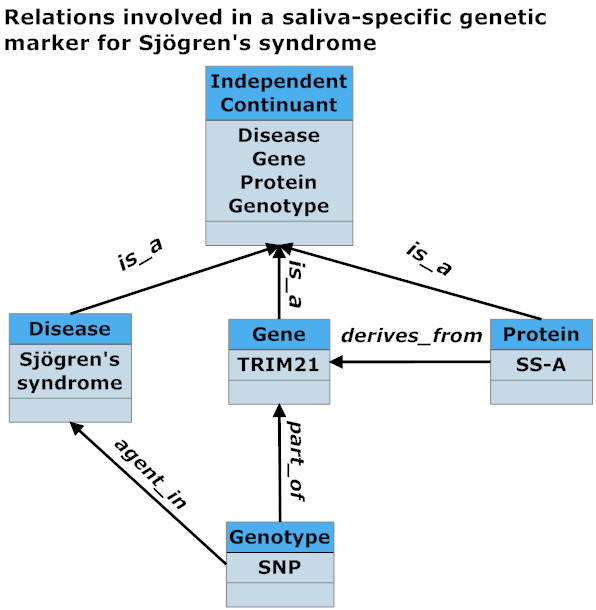
**Relations involved in a saliva-specific genetic marker for Sjögren's syndrome**.

SS-relevant portions of our ontology will be validated through our work on annotation of representative research literature on Sjögren's syndrome (including Sjögren's Syndrome Knowledge Base http://sskb.umn.edu/), through our research on the SS-A protein and on the TRIM21 gene. Figure 5, illustrates some of the relations involved in a saliva-specific genetic marker for Sjögren's syndrome.

The end-result of this work is two-fold: (1) A better understanding of relationships between diseases and multiple biomarkers because more useful information can be obtained based on the ontology annotation and classification. (2) A better aid for data interpretation, analysis and mining through the ontology annotation.

## Conclusions

Saliva Ontology is an ongoing exploratory initiative. The ontology is being constructed to interoperate with the Gene Ontology (GO), the Protein Ontology (PRO), the CHEBI Small Molecule Ontology and other standard ontology and terminology resources, including SNOMED CT. The ontology will be used to facilitate salivaomics data retrieval and integration across multiple fields of research together with data analysis and data mining. The ontology will be tested through its ability to serve the semantic enhancement of a representative corpus of salivaomics research literature that is to be included in the SKB.

## Methods

Ontology is the science of what is, of the kinds and structures of objects, properties, events, processes and relations in every area of reality [12]. As applied in the biomedical domain, ontology plays a key role in providing consensus-based controlled vocabularies serving the consistent annotation of biological and medical data and information, most conspicuously within the framework of the Gene Ontology (GO) [7] and now of its sister ontologies within the Open Biomedical Ontologies Foundry http://obofoundry.org. We believe that an approach to the analysis of saliva in terms of a controlled structured vocabulary and a common set of measurement data elements developed along OBO Foundry lines can provide a cost-effective approach to support the coordinated screening of large populations in such a way as to yield data that is capable of being aggregated for statistical purposes and for example in the context of meta-analysis.

Currently, ontologies support data integration primarily through data annotation (or 'tagging'), including the annotation of data reported in the peer-reviewed scientific literature [13]. While the value of such data annotation has been demonstrated in molecular and model organism biology and in the analysis of gene expression data [14-16], the potential of ontology-based annotation in the clinical domain has been largely unrealized due to limitations in current ontology development practices, including:

1. Most ontologies consist of only a few well-defined relations, primarily the *is_a *(e.g. heart *is_a *organ) and *part_of *(e.g. aortic valve *part_of *heart) relations, and they only relate terms within a single taxonomy [17,18]. This results in an inability to capture higher levels of biological complexity.

2. Most ontologies and terminology artifacts lack a sound logical underpinning, rest on mixed modes of classification and inadequate formal definitions, resulting in an inability to support sophisticated computation [19-24].

To address these and related shortfalls, the OBO Foundry was created in 2006 by a group of developers of OBO ontologies on the basis of an evolving set of principles designed to foster the creation of an evolving set of best practice in ontology development. The first list of ontologies satisfying OBO Foundry peer review was released in April 2010. OBO ontologies are designed to represent in an interoperable fashion the biomedical reality from which data are sampled. Their development within the framework of a common top-level ontology (Basic Formal Ontology [25]) and consistent employment of a common set of relations [10] allows Foundry ontologies to be used together as interoperable modules within an evolving larger network. The relations themselves are formalized in such a way as to ensure support for sophisticated computation both within and across ontologies [10].

We capitalize on and contribute to the OBO Foundry initiative in this work. One important element therein is the distinction between *reference *and *application ontologies*. [26]. The former correspond in medicine to the basic biomedical sciences such as anatomy and physiology. The latter correspond to the clinical specialisms and sub-specialisms, for example pediatric surgery or radiation oncology. Just as the clinical specialisms draw on the methods, theories and terminologies of the basic biomedical sciences for a variety of purposes, including the education of clinicians and the formulation of clinical research hypotheses, so, within the OBO Foundry framework, application ontologies draw on reference ontologies to serve as feeders of lexically more simple terms (such as 'protein' or 'disease') to be used in the construction of the more specialized compound terms by which the application ontologies are composed.

SALO is in this sense an application ontology. It draws primarily on four reference ontologies - Protein Ontology (PRO), Gene Ontology (GO), Chemical Entities of Biological Interest (CHEBI) and Ontology for Biomedical Investigations (OBI) - which are described in more detail below. All terms in SALO, other than those created anew because they relate specifically to the SALO domain, will as far as possible be derived from the mentioned sources. Thus for example all protein terms in SALO will be taken from the Protein Ontology. Where PRO does not have the needed terms, then requests for inclusion of these terms in PRO will be submitted to the PRO tracker [8].

### The Protein Ontology (PRO)

The Protein Ontology Consortium, led by researchers affiliated with the Universal Protein Knowledgebase (UniProt, http://www.pir.uniprot.org/), developed the PRO framework [8] with two axes of classification, based, respectively, on the protein structural units of domains, and on full-length protein sequences and their modifications. This second axis represents the various protein entities (such as splice variants, cleavage products) that can derive from a single gene.

Because proteins themselves are combinations of domains with additional sequence, the two axes of classification are related via the *has_part *relation. We are collaborating with PRO's developers in the curation of those sections of PRO relating to those proteins which are of primary interest to the saliva domain. We will also participate in PRO dissemination activities in order to expand the community of users of both SALO and SKB.

### The Gene Ontology (GO)

The Gene Ontology (GO) project is a collaborative effort to develop and use ontologies to support biologically meaningful annotation of genes and their products in a wide variety of organisms. Major model organism databases and other bioinformatics resource centers contribute to the project [27]. The GO provides a systematic language for the consistent description of attributes of genes and gene products in three key biological domains that are shared by all organisms: molecular function, biological process and cellular component. GO's value derives in large part from the fact that it has been utilized for the systematic annotation by trained biologist-curators of experimental results pertaining to multiple species of organisms and communicated in the peer-reviewed scientific literature. Some 50,000 journal articles have been annotated in this way, and their content has thereby been made accessible to computer-aided discovery. We will collaborate with GO's developers in the curation of those sections of GO relating to gene products of primary interest to the saliva domain.

### Chemical Entities of Biological Interest (CHEBI)

Chemical Entities of Biological Interest (CHEBI) is a freely available ontology of molecular entities focused on 'small' chemical compounds. The molecular entities in question are either natural products or synthetic products used to intervene in the processes of living organisms. Genome-encoded macromolecules (nucleic acids, proteins and peptides derived from proteins by cleavage) are not as a rule included. In addition to molecular entities, CHEBI contains what are called 'groups' (parts of molecular entities) and classes of entities. CHEBI includes an ontological classification whereby the relationships between molecular entities or classes of entities and there *is_a *parents and children are specified. CHEBI is available online at http://www.ebi.ac.uk/chebi/[9]. We will collaborate with CHEBI's developers in the curation of those sections of CHEBI relating to the chemical compounds of primary interest in the saliva domain.

### Ontology for Biomedical Investigations (OBI)

The Ontology for Biomedical Investigations (OBI) addresses the need for controlled vocabularies to support integration of experimental data, a need originally identified in the transcriptomics domain by the Microarray Gene Expression Data Society (MGED), which developed the MGED Ontology as an annotation resource for microarray data. In response to the recognition of convergent needs in areas such as protein and metabolite characterization, this effort was broadened to become what was initially known as FuGO (Functional Genomics Investigation Ontology) - the ontology associated with the FUGE (Functional Genomics Experiment) data model [28]. The coverage of FuGO was then further expanded in 2006 to include clinical trials and epidemiological studies, biomedical imaging and a variety of further experimentation domains, to become what is today OBI, an ontology designed to serve the coordinated representation of designs, protocols, instrumentation, materials, processes, data and types of analysis in all areas of biological and biomedical investigation. Twenty five groups are now involved in building OBI, deriving from all areas of omics research, and the Foundry discipline, including the BFO (Basic Formal Ontology) top-level framework, has proven essential to its distributed development [6]. OBI is used in our work as a source for ontological representation of biomarkers and related terms pertaining to sample collection and to diagnostic and experimental uses of saliva, as well as to associated protocols, instrumentation, statistical methods, and so forth.

### SNOMED CT

Since SALO is designed for use in support of clinical research and treatment, it is important that it be aligned as closely as possible with the SNOMED^® ^Systematized Nomenclature of Medicine, which is designed to provide the terminology needed to code the entire medical record. The current version of SNOMED is SNOMED CT (for 'Clinical Terms'), which is maintained by the International Health Terminology Standards Development Organization (IHTSDO) in Copenhagen. At its simplest, SNOMED CT is a controlled vocabulary of expressions used in healthcare reporting, as for example in an electronic health record. 'Controlled' means that the content of the terminology is designed to provide a well-managed non-redundant set of codes and associated expressions to ensure consistency of clinical coding. Quality assurance procedures are in place, which are designed to ensure that the terminology is structurally sound, biomedically accurate and consistent with current practice.

Unfortunately, while SNOMED is built around an evolving core vocabulary that is largely the work of the College of American Pathologists (CAP), it has been subjected at different times to various different sorts of combinations with other terminological resources, deriving mainly from the UK. The result is that, even after considerable efforts on the part of the new IHTSDO organization, SNOMED remains a terminology resource that is marked by multiple redundancies and associated inconsistencies of coding [29]. It is for this reason that we did not utilize SNOMED content in constructing SALO, but rather are working to ensure alignment between SNOMED CT and the results of our work on the Saliva Ontology by incorporating SNOMED CT terms where needed. At the same time, we will submit all clinically relevant new saliva-related terminology content created within the SALO framework to the IHTSDO Content Committee with a recommendation for inclusion for inclusion in future versions of SNOMED CT.

### Saliva and Ontology

No dedicated ontology has thus far been defined in direct relation to oral biological fluids [30], and the treatment of saliva in ontology and terminology resources has thus been insufficient for purposes of saliva research. SNOMED CT returns 39 records for the search term 'saliva', including 'saliva (substance)', 'normal saliva (finding), and 'saliva-induced contact dermatitis (disorder)'. Saliva (substance) is asserted in the SNOMED CT concept hierarchy to be a *digestive system fluid*, which is in turn a *body fluid*. In The Foundational Model of Anatomy ontology (FMA), saliva is a subordinate of *portion of secreted substance*; no definition is provided [17]. Given the intention of the IHTSDO to align the SNOMED treatment of anatomy (including bodily substances) with that of the FMA, and given our existing collaboration with both the IHTSDO editorial community and the FMA's developers, we will work with both communities to create, through SALO, a more detailed representation of the ontology of this bodily fluid that is optimized to meet the needs of both the clinical diagnostic community and the cross-disciplinary community of omics researchers.

Results similar to those obtained from the analysis of SNOMED apply also to other terminology resources. The Cyc ontology (which contains hundreds of thousands of terms in all domains) [31], defines Saliva is: A Type of: bodily secretion and liquid, whereby it is asserted merely that it is: An Instance of tangible stuff type.

In WordNet, saliva is defined as a clear liquid secreted into the mouth by the salivary glands and mucous glands of the mouth; it is asserted that saliva moistens the mouth and starts the digestion of starches [32].

## Authors' contributions

JA, BS and DTW conceived of the study; JA and BS designed the ontology; JA, BS and DTW drafted the manuscript. All authors made equal contributions to and read and approved the final manuscript.
